# Below the radar innovations and emerging property right approaches in Tibetan medicine

**DOI:** 10.1111/jwip.12084

**Published:** 2017-11-07

**Authors:** Harilal Madhavan

**Affiliations:** ^1^ Institute for Social Anthropology Austrian Academy of Sciences Vienna Austria

**Keywords:** innovation, intangible cultural heritage, intellectual property rights, Tibetan medicine

## Abstract

Outside the established legal framework of intellectual property rights, countries have pursued multiple pathways to protect and promote traditional medicine. As Tibetan medicine is a late entrant into commercialization, the proposals to propertize generally fall within the rationale of existing sui‐generis paradigms of Intellectual property. In this context, the article enquires the state of innovations in this sector viz‐a‐viz the property right approaches in place especially in India and China. It argues that beyond the usual complex medical science and technology led—innovations, the pathways of cumulative processes and creative additions through informal experiential learning platforms, where the transfers of knowledge become part of livelihood and social benefits (we call them “below the radar innovations”) is ubiquitous in Tibetan medicine. The trends and politics in two recent strategies of protection, that is, Tibetan medicine as economic property (emphasizing patents here among many others) and as a cultural property (intangible cultural heritage) are juxtaposed with these informal innovative attempts. The paper underlines that the productivity‐based economic rationale of these protection mechanisms should not obscure sustainability alternatives of “below the radar” (BtR) innovations in Tibetan medicine.

## 1 INTRODUCTION

The structure and dynamics of innovation in medicine often tends to be taken in isolation from the broader framework of socio‐economic systems. It is narrowly defined as science and technology driven and competition induced research on drug and devices, whose emergence is rather disconnected from the system through which health services are often delivered (Consoli & Mina, 2009). The stereotyped definition of innovation at times can create tensions and lead to disruption of the social innovation dynamics in indigenous medicines, as frequently witnessed in new organizational regulations like Good Manufacturing Practices (GMPs). Many indigenous practitioners develop and transmit knowledge from generation to generation in which individuals can distinguish themselves as informal creators or innovators, separate from the community.

Indigenous medical systems are shaped from diverse forms of interference, struggle, and creative adaptations. Some of them extend beyond one community, some are confined within a locality, some are of transnational existence, some may or may not link to the local eco‐systems, some are of migratory origin—all makes the practice, development, transmission, and ownership very curious and complex. Many new theoretical approaches within health research consider healthcare systems as knowledge economies rather than simple assemblages of technical services, personnel, and goods and institutions (Bloom & Standing, 2008; Leonard, 2002). Not only does indigenous medicine compete with biomedicine embedded in the capitalist world system (Baer, Singer, & Johnsen, 1986), but it looks beyond the operational tools that biomedicine provides in shaping up the pathways for further research and development of new medicines and challenge the protection mechanisms usually associated with the same. These characteristics of indigenous medical systems may also raise doubts about the “one fit for all” solutions of protection emerging at various international negotiation forums (Mashelkar, 2001).

For emerging industrial medicines like Tibetan medicine,^1^ any minor transformation in the process of production, organizational aspects, product variety, or elements that enable access to new or broader markets (elements of Schumpeterian conceptualization of innovation) may bring considerable impact and challenges on its development. Some of them are very “modest innovations” that are generally overlooked and ignored in official science and technology indicators. In spite of being non‐technological, they translate into substantial increase in the ability to produce and compete on a sustained basis, generating income and better living standards for those involved and create continuity in translating and popularizing the knowledge. This process of enhanced production is further incentivized upon the ability to protect and nurture those innovations in the petty‐commodity production process. These innovations will happen even if the expected economic benefits from the future are relatively low. The “exchange value” they create depends more on the ability to provide the medicines at a cheaper cost to those required patients or consumers and hence much closer to the actual “use value” of the knowledge. These innovations are well below the radar of scientific and capital investment of larger scale, which potentially create massive exchange value. These below the radar (BtR) innovations (Clark et al., 2009) are the prime movers of the Tibetan medical knowledge especially when it comes to public health of the communities they serve. For instance, in Ayurvedic medicine, social innovations and the reformulated practices known as neo‐traditional innovations offer larger possibilities in public health practices (Madhavan, 2014; Pordie & Gaudilliere, 2014).

The processes of economic globalization and transnational trade regulations obviously raise the question as to whether the international protection of intellectual property matter to “below the radar” innovations. This article examines whether and how local property right regimes govern these small innovations in traditional Tibetan medicines in China and India. The paper does not intend to discuss the familiar literature of intellectual property protection of indigenous medicines as many have written on this growing but increasingly confusing topic^2^ (Dutfield, 2005; Gopalakrishnan, 2002; Hsiao, 2007; Li & Li, 2007; Oguamanam, 2008a,2008b; among many others). Rather, this article attempts to extend this field of research to Tibetan medicine through analyzing the national policy approaches apart from the international negotiations. The interviews with Tibetan physicians from India, China, and Mongolia are used in the article along with the patent documents from published sources to understand various perspectives on innovations and policy implications in this field.

This article proceeds as follows. First, it briefly discusses Tibetan medicine, its historical origins, its makers, and its main features. Second, it explores the forms and examples of innovation investigating the concept of “below the radar innovations.” Third, it examines the way in which Tibetan medicine has been considered as a form of economic property. Fourth, it analyses how Tibetan medicine is governed at the national level. Fifth, it illustrates Tibetan medicine as a form of intangible cultural heritage. The concluding section attempts to connect these approaches and make some inferences on the inclusive innovation capabilities of these approaches.

## 2 TIBETAN MEDICINE

In its origins, Tibetan medicine is a synthesis of major scholarly traditions (ayurvedic, Greco‐Arabic, and Chinese) and the indigenous knowledge and techniques of treatment developed over many centuries by local medical practitioners. It is also called “science of healing” (in Tibetan, “Sowa Rigpa” or amchi medicine). For many centuries, it served as an important medical source in Tibet and its neighboring countries (Mongolia, Bhutan, some parts of Nepal and India and in Buddhist regions of Russia). The theory of Tibetan medicine is based on the holistic understanding of human health and its natural environment as detailed in the fundamental Tibetan medical text, *Gyüshi* (*Rgyud bzhi*). The treatment modality is predominantly pharmaceutical based but also importantly includes other practices like cupping or “moxibustion—a traditional therapy which consists of burning dried mugwort (moxa) on particular parts on the body,” as well as dietary and behavioral advice using different kinds of ingredients such as plants, minerals, animals, and metals.

The development of Tibetan medicine across Inner Asia and the Himalayas is largely entwined with the political history of this region. After a period of destruction and repression following the 1959 Tibetan uprising and the Cultural Revolution, Tibetan medicine in China was rehabilitated with state support from 1980s on wards and rapidly commercialized and modernized since the 1990s. In the Tibetan exile in India, the Tibetan medicine has been directly linked to the Tibetan nationalistic agenda as a core symbol of Tibetan identity providing a particular context for its institutional and commercial development (Kloos, 2013, 2016). In Mongolia, Sowa Rigpa was the dominant healthcare resource until 1924, when Mongolians embraced a Soviet‐style socialist reform program, which championed technological modernization along largely western lines. Soviet‐style biomedicine became the only legitimate medical system, and all other competitors were legally restricted (Janes & Hilliard, 2008). In the post socialist context of Mongolia, from 1990 onwards, the health authorities started rebuilding a system of Traditional Mongolian medicine with the state support and integrated it into the national healthcare system. Mongolian medicine is based on the same textual source (*Gyushi*) as of Tibetan medicine and hence identical theory and similar practice. In the contemporary form of Mongolian medicine, the Tibetan theories and practices have a major role.^3^

Traditionally, Tibetan medicines are collected and manufactured by practitioners themselves. Production of Tibetan medicine has evolved from manual compounding to systematic, standardized mass production in the modern industrial context. For example, in Tibet Autonomous Region (TAR) and other provinces of Tibetan regions in China, the number of physicians practicing Tibetan medicine was over 1200 in 1993, and rose to 5000 in 2010 according to official statistics (Yuan, 2012), and the industry of Tibetan medicine has grown to the extent that it becomes one of the major pillars of industrial growth of the region with 18 factories having manufacturing standards like Good Manufacturing Practices (GMP) and 300 state permitted pharmacies (Hsu, 2011). The Tibet Autonomous Region is a province‐level entity of the People's Republic of China. Chinese law nominally guarantees some autonomy in the areas of education and language policy, in practice the region is ruled by a Communist Party‐appointed cohort and hence the Chinese Health and drug policies are applicable in the region. Tibetan medical departments were attached to almost all medical institutions within the TAR and the industrial development is advancing at a rapid pace (Craig, 2011; Hofer, 2008; Saxer, 2013). The situation in countries like Mongolia and India is also quite promising in terms of increasing demand and investment of industrial capital in Sowa Rigpa. Given the expanding commercialization of Sowa Rigpa, countries have developed various rules and regulations for its protection and promotion.

Contemporary Sowa Rigpa reflects the transnational flows of Tibetan medical theory and practice, the ways in which these flows occur and the political‐economic and cultural interests which direct and transform the local practices of Tibetan healing (Janes, 2002). The globalization of Tibetan medicine may not be understood in its singularity but plurality of practice, the practice being evolved in various spaces of production such as China, Mongolia, India, and Bhutan. Tibetan medicine is also expanding globally. We need to wait and see in which directions Tibetan medicine unfolds in the West. In fact, questions arise as to whether in Western countries it maintains its traditional meaning as a theory and practice of every day wellness, or whether while consciously carrying the name of the ancient tradition, it is being transformed through markets (Banerjee, 2009) into something quite different from its original version. This will essentially invite the familiar challenges of its legitimation through parameters of biomedicine and the larger political economic issues that the other indigenous systems have already been through in the course of their transnationalization process (Pordie, 2010; Reddy, 2002). This may lead to conceptual shrinkage and limit to therapeutic innovations within Tibetan medicine as in the case of Ayurveda (Banerjee, 2009). This may overlook the inherent heterogeneity of practice as assumptions of homogeneity of biological bodies are the very rationale for any pharmaceutical expansion.

## 3 BELOW THE RADAR INNOVATIONS (BtR) IN TIBETAN MEDICINE

In China, the embracing of neoliberal economic principles in pursuit of economic modernization has led to a positioning of traditional medicines including Tibetan medicine in view of the global marketplace. In this “new pluralism,” economics has become the dominant discipline in global health (Cant & Sharma, 1999; Janes, 2002), and initiatives like “health for all” have been replaced with narrow valuations of cost efficiency, dominated by private interests leading to increased user fees and other charges in public healthcare and hence increased cost of pharmaceuticals. The economic evaluations of Medical efficacy is increasingly defined as health gains per dollar spent (the gained life days calculated in quality‐adjusted life years [QALY] and Disability adjusted life Years [DALYs]) and conflated with market efficacy. Hence, therapeutic pluralism has become very competitive and the market subsequently decides the choice and allocation of resources. While the neoliberal health interventions are based on the said definitions, Tibetan medicine need to be efficient in dealing with potential public health challenges, which in turn requires effective medicines, basic research and development and market potential.

The ways in which some of the Tibetan medical formulas are reaching the global market and the private initiatives are remarkable. Though we have examples of Tibetan medical formula like Gabur‐25 (for circulatory disorders such as atherosclerosis) reaching the global market as a modern pharmaceutical product abiding all the modern European regulatory guidelines, it is not very common to have such path‐breaking innovations with classical formulas in a very competitive pharmaceutical field (Schwabl & Vennos, 2015). This is because Tibetan medicine integration into the Western regulatory framework, which only works for formulas, composed of herbal and mineral substances, while the inclusion of any kind of animal components makes the product registration arduous. This calls for compromise in its terms of substitution. In the meantime, there are many innovative experiences operating below the market and policy radar with in this medical system, that is pioneering and disruptive, which constantly adapt to the local conditions, and add substantially to the development of the sector and bring transformations to the life of those live with it. Moreover, they address the social and environmental concerns that public policy is usually struggling to tackle.

Some of these innovations are able to make a larger impact on the market, while some others are able to provide better health options to the public and in some cases, both. These may include products such as medicines, teas/nutraceuticals/food supplements. Local companies such as the major Tibetan pharmaceutical manufacturing entity Men‐Tsee‐Khang (MTK) in Dharamsala reformulate the traditional recipes of Tibetan knowledge using the *Gyushi*, as the basic reference point.^4^ The resulting herbal products include not only anti‐aging cream, anti‐fatigue teas, and skin care products but new medicines to address liver failure, mental fatigue, multiple sclerosis, and hepatitis. Some of them are reformulations and many others are even change in content. A Physician from Dharamsala, the second capital of Himachal Pradesh state, in India mentioned that;

These are new pills. So the text still gets updated. We test these pills with the patients. But there's no danger, because they are based on good knowledge. At first, we make only small amounts, and then we see if they work well or not …. Now it's different from before: If we make Agar 35 [a lung medicine], we make large quantities of it, it goes to the entire world—India, Europe, America, and so on. Before, the medicines could be individualized according to the patient's condition and also according to the environment, the climate … Now, we have to think “general.”^5^

Multiple types of innovations are prevalent in this sector. One example is the innovation of medicines through simple alterations to fit the geographical locations. In the old texts, the quantity of Aconitum (which itself is poisonous) in the formula for khyung‐Inga (The prescription is used for diseases caused by worms, heat disorders, skin diseases etc.) is very high (informed by the personal interviews). Now in India, many physicians mention that they use much less khyung‐Inga in their medications so that the patients will not faint. They also noted that Indians get dizzy more easily because of their diet in contrast to Tibetans. Now, since most branch clinics of MTK cater mostly to Indian patients, the institute took a conscious decision to consider this difference during the production process. Many physicians from Dharamsala noted that they use the old Tibetan texts to do their own research for finding the new combinations and establishing the efficacy of the existing ones.

While demand for Tibetan Medicine is on the rise, there are supply constraints. Both locals and tourists demand Tibetan medicines; questions have arisen as to whether better packaging and format (pills and powder) would facilitate their trust. For example, a popular physician from Ladakh (India) mentions:

I would put English and Ladakhi on the packet. Demand will increase when patients know what is inside the medicine. If I could pack powders in this way, then the National Rural Health Mission (NRHM—the government health functionary) would supply them to the public hospitals. They need something that lasts long time, is easy to prescribe and easy for patients to take. If I show this packet, they will surely ask me to bring it. This could be the next step that will bring me success.^6^

He thought that transparent information about the product is also relevant. At the same time, he mentioned that any kind of innovation is expensive and for the individual physicians it is difficult to manage within the limits of their earning.

The above conversations narrate some of the evidences for non‐patented but meaningful innovations in Tibetan medicine. Although such innovations in Tibetan medicine may fall well below the radar of scientific innovations and existing patent requirements, without doubt, they have larger repercussions in crafting a niche for Tibetan medicine and its practitioners. These inexpensive but effective frugal innovations (below the radar) may be imperfect, but have a wider outreach and are not limited to low‐resource settings: ingenuous ideas can be adapted to offer simpler and efficient alternatives to mainstream care. This is important as many international organizations justify the promotion of indigenous medicines mainly on economic grounds like low‐cost access to medicines and universal health coverage (WHO, 2002, 2013; WIPO, 2013). In this process, the national and international health policies tend to equate the “medical” space of traditional medicines to that of biomedicine. Yet, it is important to mention that Tibetan medicine has been able to maintain somewhat more conceptual and epistemological autonomy than other Asian medical systems (e.g., Janes, 1995, 1999) in this phase as it is even now being mostly practiced by the traditional physicians and less commodified in comparison to other Asian medicines. After having illustrated the notions of Tibetan medicine and below the radar innovations within the same, the article now considers conceptualizations of Tibetan medicine as an economic property (section 4) and, after a scrutiny of Chinese and Indian laws governing the same (section 5) as a form of intangible cultural heritage (section 6).

## 4 TIBETAN MEDICINE AS ECONOMIC PROPERTY

This section examines the conceptualization of Tibetan medicine as an economic property in the world pharmaceutical market. Of many factors, the increasing cost of drug discovery and off‐patent debacle of 2012–2015^7^ and the consequent exigency for herbal formulations made many pharmaceutical companies to renew their strategies in favor of indigenous knowledge based drug development. Rausser and Small (2000) pointed out that the contribution of traditional codified or non‐codified medical knowledge largely enhances the probability of drug development at the lead discovery stage (sometimes as high as 50% chance of success), and thereby reducing attendant costs and time taken for the initiation of the drug development process. Although critics suggest that the chemical complexity of natural products may make commercial production expensive or impossible (Firn, 2003), bioprospection is often used as a pertinent direction of research in many pharmaceutical companies.

Among codified systems, Chinese medicine and Ayurveda (a system of traditional medicine with historical roots in Indian subcontinent), are more commercialized and frequently subject to bioprospection (Patwardhan & Mashelkar, 2009) and at the same time victims of repeated cases of biopiracy too. The Traditional Knowledge Digital Library or digitalization of traditional knowledge of India is launched to protect the knowledge from patent biopiracy especially in the field of medicine. It is on record that the Council of Scientific and Industrial Research (CSIR) successfully revoked patents filed on turmeric, *Neem* and *Basmati* in USA. In the case of neem, it is important to underline that the patent was only subsequently revoked and that the patent holder was able to exploit its monopoly until 2000 from 1994. No biopiracy case related to Tibetan medicine (Sowa Rigpa) has been reported yet, which does not mean that it is not a target for bioprospection. Mostly, however, transnational corporations are interested in single plant formulations to extract the active ingredient, while Sowa Rigpa consists mainly of multi‐compound formulas whose biochemical mechanisms are too complex and hence expensive to investigate. Tibetan medicine often recommends complex herbal mixtures and multi‐compound extracts. It advocates for a polypharmacological approach to deal with multifactorial causes such as chronic and degenerative ailments. Hence the concept of “one disease—one target—one drug” does not hold true for Tibetan medicines.

Sowa Rigpa is at a nascent stage of industrial development compared to Ayurveda and Chinese medicine, but a number of firms have started its mass production, mostly Tibet based companies in China but also in India, Mongolia, Inner Mongolia, and Switzerland. There are some initial attempts to map and narrate the modernization, institutionalization, and industrial formation of this system in specific areas (Blaikie, 2013; Kloos, 2013, 2015; Saxer, 2013). Even though in indigenous medicines, the actual expenditure on research and development (R&D) is considered to be less than the mainstream pharmaceutical sector (Madhavan, 2011), now there is an increasing attempt by larger pharmaceutical corporates to invest in R&D on herbal formulations. As a result, patented medicines, either through active compound research or reverse engineering are of high priority in Chinese medicines and Ayurveda. In this context, the Tibetan medicine with its huge volume of unexplored knowledge can potentially be treated as an economically tradeable commodity and hence patenting is obviously considered as an indicator for increasing research attempts and a prevailing form of assurance to market profitability.

This article has searched evidence of the use of Tibetan medicine in patents by searching through google advanced patents, where the information originate from the United States Patent and Trademark Office (USPTO), the European Patent Office (EPO), and the World Intellectual Property Organisation (WIPO). Google Patents covers the entire collection of filed or granted and published patent applications from the USPTO, EPO, and WIPO. The US patent documents date back to 1790, EPO and WIPO to 1978. I have used the key words like “Tibetan medicine” and “Mongolian medicine.” The search for Tibetan medicine in this interface shows that there are around 2300 applications (till December 2015) either granted or filed, which have references to various Tibetan medicine formulas or use in Tibetan knowledge. A search with the keyword “Tibetan medicine” alone gave a result of 1589 observations. Interestingly most of them are filed in the last decade. The number of granted applications is listed in Table 1.

**Table 1:** Number of patents issued by various patent offices on products/processes that has reference to Tibetan medicine until December 2015

Patent offices	Patents
China	330
USPTO	40
European patent office	7
Germany	3
Canada	1

*Source*: Compiled from Google Patents.

What is important is that a large number of patents are applied and awarded in Chinese, US, and European patent offices, which claimed a close or remote reference to Tibetan formulae. This, prima facie, underlines the increasing attempt to claim the property ownership over many formulas in Tibetan medicine. How many have been actively converted into the industrial application is not evident. But this can lead to restricted use of even the traditional formulas and for sure lead to further drug development or bioprospection possibilities and hence it may change the course of the industrial structure from a very petty commodity, proto‐industrial system to a monopolistic competition. This has a critical impact on the property rights systems within the sector as well, as many of these home‐based physicians’ informal innovations might be challenged by the new industrial ownership claims.

The new European directive 2004/24/EC for traditional herbal medicinal products that followed from the registration methods in Austria (HERB‐00037) and the UK (39568/0001) and the success of ethnopharmacological based preparation within a modern Western medical and regulatory framework like Gabur‐25 (Schwabl & Vennos, 2015) may work as inspiration for many further drug development cases. The 2004 European directive replaced the earlier directive, which required documented scientific evidence for the efficacy and safety of all pharmaceuticals, including herbal medicines. The new directive ensured the existence of herbal medicinal products (HMPs) and agreed to consider particular characteristics during the assessment of their quality, efficacy, and safety. This has defined two categories for herbal medicines: (a) well‐established use HMPs, which can be granted a marketing authorization; and (b) traditional herbal medicinal products, which can be granted a registration based on their long‐standing safe and efficient use. The second category is a new entry that accepts the documentation proving long‐term traditional use. Still, the clause that suggests 15‐year use in any European member states creates difficulty for many traditional medicines for European market entry.^8^

Tibetan medical preparation for treating hyperlipidemia (patent publication No. CN102698185B) pharyngitis (CN103301271A), conjunctivitis (CN103316085A), alcohol withdrawal medicines (CN102058849B) anti‐inflammation and pain relieving medicines (CN102716277B), kidney disorders (CN102579818B), cervical spondylitis (CN102085339A), rheumatism and rheumatoid diseases (CN100427062C), haemorrhoids (CN103313116A), cerebral thrombosis (CN103330858B), gastric ulcer (CN102846729B), etc., are some of the examples of the patents filed in these offices (For additional details, see appendix 1).

Most of these patent applications are filed by Chinese corporates and even most of the patents on “Mongolian medicine”—another search term we have used—also emerge from these groups. A path dependent development strategy of traditional Chinese medicine (TCM) is apparent in China's policies towards Tibetan formulas. The major disease categories in which the medicines fall in are diabetes, peptic ulcer, hyperlipidemia, kerato conjunctivitis, influenza, cough, hypertension, chronic bronchitis, hemorrhoids, pharyngitis, etc., and the patents are claimed by the researchers or corporate institutions, which mostly are part of the production units in China include both Tibetan and Chinese owners.

The increasing patent applications by the corporate manufacturing may not only exclude actual custodians within the Chinese Tibetan territory but may also pose questions as to the ownership and practice of Tibetans living outside China like Tibetan in exile and those who practice Mongolian medicines, etc. In the phase of corporatization of indigenous formulas, this remains as a major concern. For example, in 2000, the Council of Scientific and Industrial Research (CSIR) in India conducted a study and found that 80% of the 4896 references of individual plant based medicine patents in USPTO that year related to just seven medicinal plants of Indian origin. Three year later, there were almost 15,000 patent filings related to Indian herbs and medicinal plants across the USPTO, European Patent Office (EPO), and other global patent locations. In the view of CSIR and other domestic Indian R&D entities, several of these patents encroach on prior art documented in ancient Indian medical texts (Choudhury & Khanna, 2015). Hence, one of the big concerns is the granting of wrong patents to the multinational corporations, while various communities and individuals practice the same knowledge for years.

**Figure 1:**
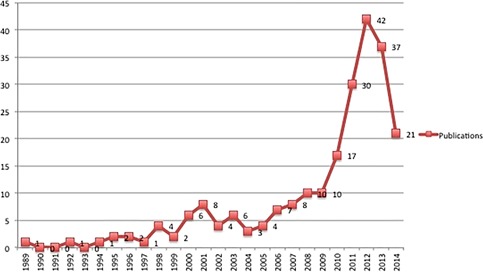
The number of clinical trials published, 1989‐2014 (Luo et al., 2015)

While patents remain as a foremost indicator that visualizes Tibetan medicine as an economic property, the second is increasing number of Randomized Control Trails (RCTs) in Tibetan medicine research (see Figure 1). One recent research found that a total of 227 RCTs involving 29,179 participants were reported in China, which differs in terms of study size, sites, treated conditions, interventions, measured outcomes, and quality (Luo et al., 2015). According to the study, 103 diseases or symptoms were reported in the included trials. Medical interventions used in the RCTs consisted of drug treatments and non‐drug treatments including bloodletting and moxibustion in which Tibetan patent medications for oral use were tested in 175 studies and for external use in 47 studies. 93.8% (213/227) of the trials reported exceptional efficacy of Tibetan medicine over control interventions. Fifty‐three percent of the clinical trials were conducted in Western hospitals. Publication numbers fell in 2013, possibly due to the decrement of RCTs testing single Tibetan patent medicines, and most of which were products of a single company (Cheezheng Tibetan Medicine Co., Ltd).

Another related study gives important information about the clinical trials in Tibetan medicine done in the West (Reuter, Weisshuhn, & Witt, 2013). The study notes that there are compromises done on the traditional Tibetan compounds for adaptation to the local situations and regulatory aspects, which forced the reinvented formulas to have lesser components (e.g., the case of traditional Byu‐Dmar 25 to Byu‐Dmar 13 [medicine for migraine prophylaxis] (Schwabl & Vennos, 2015)). But most of these studies are underreported as information on clinical research published in Tibetan is not available in indexed journals.

The adaption to globalization and free trade force a major shift in Tibetan medicine both in terms of ownership rights and in evidence creation through patentability and randomized controlled trials. This process may eventually fit into the same discourse of pharmaceuticalization like in the case of other Asian medicines (Banerjee, 2009) and may limit the possibilities of Below the Radar (BtR) transformative attempts and may redefine the rights ignoring its heterogeneity in practice. These implications may not directly follow or translate from the international property right regime, but largely depend on how the national policies are advocated within the broader framework of global IPR rules. This article uses China and India's regulation of Tibetan medicine as useful case studies. The next sections will explore various forms of property right approaches adopted by India and China for the growth of their respective industries.

## 5 SUI‐GENERIS ATTEMPTS IN TIBETAN MEDICINE—CHINA AND INDIA

Trade related Intellectual property Rights have provided signatory countries the freedom to choose intellectual property protection either under a patent regime or sui generis system or a combination thereof, hence different countries follow different approaches in terms of protection of traditional medical innovations. A combination of IP protection is in use in China—which is not equivalent to patent protection but rather a system that permits two types of protection mechanisms. A sui‐generis law is applicable to those preparations, which are not covered under the provisions of patent law, but listed in the national pharmaceutical standards. It is administered by the Health Department and the regulations cover final products, extracted substances and their preparations of Chinese and Tibetan medicines. The criteria of novelty and inventive step (which are important clause for patent approval) are relaxed to an extent in Sui generis—with a first class protection with long (10/20/30) years of protection and second class with short (seven) years of protection. The first class include those formulations that (1) having *special curative effects* for a certain disease; (2) *artificial medicines* prepared from varieties of wild medicinal materials; or (3) used for the prevention and cure of *special diseases*. The Second class protection may be applied for (1) conforming to the provisions of first class in these Regulations, or having once been listed under first class protection but now being cancelled; (2) having outstanding curative effects for a certain disease; or (3) effective substances and special preparations extracted from natural medicinal materials.

Saxer (2013) mentions that around 1,257 Tibetan medicines were under protection in the second category in 2009 and 12 of them in the first category. The second class provisions also included some traditional formulations that is already there in the public domain.

The Decree No. 106 of the State Council of the People's Republic of China^9^ says that:

*Article 16*: The period of protection of types of traditional Chinese medicine under second protection may be extended for seven years upon expiration. If it is necessary to extend the period of protection of a type of traditional Chinese medicine under second class protection, the producing enterprise shall, six months before the expiration date of protection, submit an application for extension according to the procedures….

*Article 17*: The production of protected types of traditional Chinese medicine within the period of protection shall be restricted to enterprises, which have been granted the Certificate of Protection of Types of Traditional Chinese Medicine, unless otherwise provided for in Article 19 of these Regulations.

And article 19 gives special permission to the certified firm to allow/license other firms to duplicate their medicines with a certain mutually agreeable amount of royalty.

However, the exclusive right of production of traditional formulations to some companies in the second category led to a legal dispute within China itself. So called precious pills^10^ like Rinchen Ratna Samphel, Rinchen mangjor and Rinchen Drangjor were allowed to be produced only by TAR Tibetan medicine factory in Lhasa and Arura group in Xining. This, in turn led to a major fight between Dr. Jigme Phuntsog (An influential lama of the Nyingma tradition of Tibetan Buddhism and owner of the second largest Tibetan medical company in Xining) on the production rights of the first two precious pills by Arura group for 14 years with a renewed second‐class protection. Dr. Phuntsog questioned the government and defended his fundamental right of practicing the monastery learned knowledge, but failed in the legal attempt. This showed that the state mechanism to provide the exclusive trade rights for classical products to one particular company actually entered into the rights of the many who have experience in practice and making of the same medicine.

In China, formulas officially documented and locally registered before 1997 are granted the status of “old” knowledge. All other oral and textual knowledge is treated as a new knowledge and not listed in the Tibetan medicine standards and Chinese pharmacopeia until they are proven through comprehensive scientific studies. In reality the distinction is not between new and traditional, but between already documented, filtered, and approved knowledge and that which is yet to undergo that process (Saxer, 2013). The fact is that the window of undergoing the process of documentation was kept as low as possible and hence large part of oral knowledge is obliged to produce new scientific evidence to enter the pharmaceutical market. The scientific research necessary for this registration costs between $300,000 and $1.4 million per drug.^11^

This complexity in the property assignment and imbalance in the power relations is of serious concern. The regulatory requirements in China go beyond what is required in India and in Europe. Hence, many formulas are not locally registered and officially documented, fall outside the Tibetan drug standards and subjected to increasing pressure to comply with Chinese state food and drug administration and GMPs. As the patent paradigm enjoys the expediency of an internationally established legal protection and recognition, it is understandable that China is increasing the share of patent protection with in Chinese and the Tibetan medicine sector, while the other portion of domesticated sui‐generis applications are shrinking over the years.

In China, patents are also used in an effective way to improve the shortcomings of the traditional formulations as well. They actually help in overcoming the “incompatibilities” of ancient preparations for the market, for example, the *Huoxiangzhengqi* liquid, even though very effective, but the taste was difficult for many patients due to the residual alcohol. The technology of Patent No. 91107254.3 effectively solved the problem of high amount of ethanol in the oral liquid (Saxer, 2013). *Bunao jianshen* pill, as one of the classic ancient prescriptions in *Shandong*, was used for brain‐strengthening and Kidney‐nourishing, but it had the problem of market promotion because its cinnabar coating contained heavy metals like mercury. The technology of patent No. 94110752.3 had solved this problem of cinnabar coating.

Since the Tibetan medicine in china is driven by the policies applicable to traditional Chinese medicine, it is interesting to note the definitions of various terms used in the patent clause. Under article 25 of the Chinese Patent Law 1992, traditional medicine can be protected through three categories—products, usage and methods. Under “products” the following can be covered: TCM prescription, herb, relevant product, medicinal compound with its active compounds. Under “usage” new medical use can be covered while under “methods” processes like extracting, preparation, and formulation can be covered. Thus, both product patents and process patents are available for protecting TCM. Under the Article 22 of Chinese Patent Law, the criteria are defined as:

*Novelty* means that, before the date of filing, no identical invention … has been publicly disclosed in publications in the country or abroad or has been publicly used or made known to the public by any other means in the country (not abroad), nor has any other person filed previously with the Patent Administration Department Under the State Council an application which described the identical invention and was published after the said date of filing. Inventiveness means that, as compared with the technology existing before the date of filing the invention has prominent substantive features and represents a notable progress. Practical applicability means that the invention can be used to produce effective results (PRC, 2008).

Besides the above criteria, applicants have to provide full description of the invention (disclosure of methods but not origin) so that anyone skilled in the art is capable to carry it out.^12^ From the above statements, it is evident that the knowledge in use outside the country could be patented in China, as novelty clause is defined in a very narrow sense. Since only methods are revealed and not origin, the question of prior informed consent and details of appropriation may remain hidden. These definitions in the patent regulations may give ample room for illegitimate appropriation of knowledge in Tibetan medicine practiced outside of China.

In India, Tibetan medicine was accepted in 2010 within the legal framework of Indian medical systems and hence the production regulations are expected to be placed under the Drugs and Cosmetic act (DCA) 1940, with minor amendments. The department related parliamentary standing committee on health and family welfare of Government of India stated that^13^:

The Committee observes that keeping in view the distinct nature and functionality of Tibetan medicines, the same should be defined separately with specific provisions for their regulation, surveillance and monitoring under the scope of the Drugs and Cosmetics Act and Rules on the pattern of other Ayurveda, Siddha and Unani drugs (AS&U) from the outset. The Committee, therefore, recommends that suitable modifications may be made to the Drugs and Cosmetics Act and Rules (para 6.16).

In India, a sui generis system was set up to provide grassroots innovators an incentive to disclose knowledge and origin (GoI, 2002). AYUSH department have been funding for many documentation efforts on different systems of medicines including Sowa Rigpa. A pharmacopeia compilation has also been initiated in India. The system in India is different from China as the DCA recognizes three categories of protection like patented medicines, proprietary medicines, and classical medicines separately with different regulations. The Tibetan medicines are yet to be defined in the same way, but the process is underway.

DCA defines the classical drugs as:

Ayurvedic, Siddha or Unani drug includes all medicines intended for internal or external use for or in the diagnosis, treatment, mitigation or prevention of [disease or disorder in human beings or animals, and manufactured] exclusively in accordance with the formulae described in, the authoritative books of Ayurvedic, Siddha and Unani (Tibb) systems of medicine], specified in the First Schedule (with an amendment in 1982)

Proprietary medicines as:

In relation to Ayurvedic, Siddha or Unani Tibb systems of medicine all formulations containing only such ingredients mentioned in the formulae described in the authoritative books of Ayurveda, Siddha or Unani Tibb systems of medicine specified in the First Schedule, but does not include a medicine which is administered by parenteral route and also a formulation included in the authoritative books as specified in clause (a) (GoI, 1940).^14^

In the first case of classical (*shastric*) drugs, no change in the name of the products, ingredients, indications are allowed, proof of efficacy is not required and should not be advertised in public media while the second type can have change of the combinations, but the ingredients should be mentioned in the text and can have brand names along with product names, proof of efficacy is required and can be advertised through public media. In India, there are ambiguities regarding the approval for proprietary medicines as it is filed and approved through the district officers, and state drug controllers and state ISM directorates. With small number of staff in these departments, how efficiently the proof of efficacy and the prior art search are done is still a matter of concern. However, what I intend to emphasize here is, the Indian regulatory system at least provides a room for traditional formulations to thrive and a system in place to promote the grass‐root and family based innovations/formulations.

Section 6(i) of the Biological Diversity Act of India, 2002 (GoI, 2002) and the Patents Act, 1970^15^ requires an applicant to obtain the necessary permission from the National Biodiversity Authority before applying for a patent for any invention based on biological resources obtained from India. The process of granting such approvals by the National Biodiversity Authority is carried out in consultation with the State Biodiversity Boards, if necessary. Even though the ownership of the State over the biological resources and the difficulties it poses to the Ayurvedic firms being questioned (Agarwal, 2001), now the traditional knowledge digital library (TKDL) and biodiversity management committees (safeguarding and managing the documentation the knowledge in different panchayats through biodiversity registers) has become an integral part of effective monitoring system of traditional knowledge innovations.

The reverse engineering and phyto‐medical approaches in drug discovery has impelled the process of herbalization and alienation from its epistemological roots, but only projects its market efficiency through a property rights system. Tibetan medical formulas (which largely rely on herbs, minerals, and animal ingredients) now may be projected as a part of herbal system with US or European patents to tap the global market for “natural” medicines. The patent statistics might be an indication towards the same. Strict scientific approach stripping its theoretical plurality would win the markets for herbal medicines as seen in the success story of Japan and Germany as leading suppliers of herbal medicines and botanical medicines (Hsiao, 2007).^16^

## 6 TIBETAN MEDICINE AS CULTURAL PROPERTY

The 1970 UNESCO convention prohibit and prevents the illicit and illegal import, export and transfer of ownership of cultural property.^16^ These obligations called for States “to protect the cultural property within [their] territories against dangers of theft, clandestine excavation and illicit export.” States are asked to undertake practices with whatever means they have at their disposal to assist in making necessary reparation, and to prevent the illicit trade and transfer. The convention provides for States to designate items that are of cultural importance. These items have to be of importance in archaeology, prehistory, history, literature, art or science, rare collections and hence naturally, the specimens of fauna, flora, minerals and anatomy, and objects of paleontological interest are protected. The Convention allows source nations to create an institutional structure for protecting their cultural property and define “illicit” in any terms they wish, and Member States must enforce the export laws of foreign States (Veres, 2014). The concept of cultural property has been successfully used at the national level for protection of Tibetan medicine in China.

The increasing concern of globalization and the threat it may pose to the traditional cultural expressions (TCEs) and popular culture culminated in a mechanism to safeguard TCEs at the international level. According to the UNESCO Convention for the Safeguarding of Intangible Cultural Heritage (ICH),^17^ traditional healing also forms a type of intangible cultural heritage, which is defined as;

Practices, knowledge, skills—as well as the instruments, objects … associated therewith—that communities, groups … and individuals recognize as part of their cultural heritage. This intangible cultural heritage, transmitted from generation to generation, is constantly recreated by communities and groups in response to their … interaction with nature … and provides them with a sense of identity and continuity, thus promoting respect for cultural diversity and human creativity.

Specifically, the convention mentions that healing may fall in the section of knowledge and practices concerning nature and the universe of ICH;

This domain includes numerous areas such as traditional ecological wisdom, indigenous knowledge, knowledge about local fauna and flora, traditional healing systems, rituals, beliefs, initiatory rites, cosmologies, shamanism, possession rites, social organizations, festivals, languages and visual arts.^18^

This approach has inspired China to create a national list of intangible cultural heritage. The country has a strong institutional infrastructure including the Intangible Cultural Heritage Protection Centre of Chinese National Academy of Arts (CNAA), which takes full advantage of the available academic resources to evaluate the potential ICH candidates, prepare and organize relevant for the ICH list. Apart from this, Chinese Government have established a “National Expert Committee for Protection of Intangible Cultural Heritage,” which is composed of 68 specialists who make suggestions on the planning of conservation programs, implementation of plans, evaluation on the name list and successors of the ICH at the national level. From 2004, a vast inventory work was undertaken, leading to the first official listing of China's national heritage in 2006 (518 elements), then the second in 2008 (510 elements), and a third in 2011 (191 elements). In addition, 1,219 elements have thus been recognized at the national level so far. At the same time, the provincial heritage projects amount to around 8786 projects, which is huge in terms of detail and scope. In 2009, the Chinese Central Committee and the State Council of China had jointly issued a number of important documents, which established principles of ICH protection initiatives in China and determined the “Level 4 protection list system,” which included national level, provincial level, city level, and country level protection elements. The system included naming, awarding, commendation, reward, and other ways for protecting ICH and representative inheritance persons in China.

Tibetan medicine was one among the nine medicine‐related cultural heritages^19^ created in 2008, and so far medicine including Chinese, Tibetan, and Mongolian medicine forms 3.7% of all Chinese intangible heritages (Ye & Zhou, 2013). But a close analysis of the document of Tibetan medicine in the Chinese intangible heritage application shows that the main Tibetan treatise, *Gyushi* is mentioned only in passing in the last paragraph of the English version (aimed at the international readers) and the Chinese version is presented in a much different way and highlights techniques of mercury processing in Tibetan medicine (Obringer, 2011). Gerke (2013) and Saxer (2013) mention that *Tsotel* is a detoxification method, a creation of mercury—sulfide ash, a key ingredient of almost all precious pills in Tibetan medicine (now Lhasa Men‐Tsee‐Khang has a state issued patent from 1990s) also figured in the ICH list of China in 2006. This protection may run into conflicting ownership for multiple stakeholders of the same tradition, but it may remain harmless for others, as national ICH jurisdiction is within the nation state.

Out of the national heritage list of medicines, both acupuncture and moxibustion are accepted in the *Representative list of the intangible cultural heritage of humanity* in 2010. But, Article 3 of the ICH convention adds that nothing in the Convention may be interpreted as affecting the right and obligations of states parties deriving from any international instrument relating to IP rights or to the use of biological and ecological resources to which they are parties (Vadi, 2007). Considering knowledge as a “public good,” unlike the biological resources, its shared use need not be curtailed or confined. Simultaneous use of a public good like knowledge either can improve the ways of utilizing the knowledge or can find a better use instead of “tragedy of commons,” which can happen in the case of biological resources. So as a sustainable protection mechanism, the use of one country's cultural heritage application for a traditional knowledge, normally should not affect the right to use this knowledge by other country that shares the same cultural heritage. It can create some tensions or conflicts among those nations who shares a type of ICH. The engagement of local communities in these applications needs to be ensured by the nominating nations.

Many plants and natural products of the Tibetan pharmacopoeia are either endemic or already limited to certain protected areas. This raises the question of how an increased demand for such products would be handled. Providing ICH rights to any particular country less likely to hinder the free flow of resources, as article 3 of the ICH convention mentions that the convention does not alter the obligations under the other international law instruments such as TRIPS agreement (Vadi, 2007), unless economic sanctions are forced upon the traded materials. This is possible either due to an increasing pressure for raw material protection or due to political conflicts regarding the ownership of knowledge. China proposes a “Productivity‐based Protection,” that is the ICH and the resources thereof are transformed into products through manufacturing, circulation, and distribution, so that the economic benefits are available and so the development of related industries is possible. They argue, like many others, that the ICH can be positively protected through production practice and a favorable interaction between the intangible cultural heritage and the coordinated social and economic development can be achieved.^20^

Protection of ICH also has implications in terms of researchers’ freedom to deal with the ICH categories. Foreigners will need approval from the provincial level authorities before they can conduct any surveys involving ICH and such research must be done in co‐operation with Chinese research institutions, and reports must be made on the results. Copies of field notes and pictures must also be submitted to provincial authorities. The individual researchers who violate the rules may be subject to fine about US $1500–7600, while the organizations may be fined ten times of this amount. Some sort of monitoring also holds true with biological diversity rules in India. It also states that the resources would be owned by the State and any industrial usage needs two levels of permission from the national biodiversity authority and the state management committees. The biodiversity rules in India have already raised the eyebrows of the manufacturing associations of Indian medicines, as it poses multiple hurdles in accessing the resources. There is no doubt that the economics of these cultural resources attains more attention than ever before.

What I argue is that there is an economic intention behind most of the protection possibilities, either through existing intellectual property coverage or through the ICH approach. In the neo‐classical economic model, even though the patent monopoly is justified by benefit to the public, it alters the competitive environment between the market players. The winner takes the entire market outcompeting the established market actors, which allows the market powers to consolidate their power through new forms of research. On the other hand, the innovations in Tibetan medicine are of different genre. These cumulative innovations include improvements in existing medicines, finding new processes that are not covered by patents, compounds with slightly different properties or new forms, mostly added through experience and public feedback. Such innovations may fail to meet a high threshold of inventive step, as the invention is not of technological nature beyond the prior art. Nevertheless, in a situation where there is no product patent or where the length of the patent is shorter, such innovations are used to bring in cheaper and effective generic equivalents (Srinivas, 2013). How far local resistance can change the fate of negative impacts of global property right regimes is doubtful, though Pordie (2008) argues that in the Ladakh Himalayan region, although the local protagonists agree to follow the protection policy, they redefined its meaning and purposes in such a way to serve their interests on the level of their community, reaffirming their ethnicity and medical identity. Such kind of local alternatives needs to be empowered.

A significant number of Tibetan medical practitioners have been ignored under the category of local health Traditions with no public support and regulations, while they remain in high demand due to the low cost but effective healthcare options. The local technological capability of many small medicine producers is not adequate enough to either effectively implement the new regulative structures or complete in the commodification phase. Healthcare policy researchers argue that a strong national capability for both technological and social innovation in developing countries represents the only truly sustainable means of improving the effectiveness of health systems (Gardner, Acharya, & Yach, 2007). Benefiting from this commodification depends not only on the availability of legal rights that are enforceable beyond the locality, but also on the ability of traditional knowledge holders to take advantage of the national and international law including property and access rights relating to the resources, land, and intellectual property (Dutfield, 2005). The last two sections showed that how effectively China has developed an institutional infrastructure for protecting their cultural property while India has enacted the sui‐generis laws to make the traditional formulas and innovations inclusive within the existing property right framework.

## 7 CONCLUSION: GLOBAL PROPERTY RIGHTS AND WITHERING BtR INNOVATIONS

Economic liberalization and the transnational movement of knowledge offer multiple possibilities. The increasing number of patent applications and clinical trials show that the Tibetan medicine has already made inroads into the global pharmaceutical market. The Countries like China have been utilizing multiple property right mechanisms to provide incentives for better investments in this field. A path dependency to Chinese medicine and productivity based promotion is certainly justifiable on economic grounds within the national boundaries, but as a trans‐cultural heritage, Tibetan medicine stakeholders may benefit more from incorporating the traditional formulations in new protection mechanisms. Moreover, it may be argued that the knowledge holders and practitioners are bound to become victims of the vagaries of market forces and seldom receive a fair percentage of the value additions realized through commercialization due to their weak bargaining power and limited social and technological capital.

While the property rights system based on patent protection and heritage preservation are rapidly emerging in different countries and have acquired international significance, what Tibetan medicine needs to negotiate is a space for frugal innovations. The race for scientific validation and the exorbitant cost involved in it will force local innovators and physicians into non‐negotiable trade relations, forced ownership contracts, and comparatively disadvantaged research partnerships. The established patent system does not recognize “new methods” in “diagnostics” and “treatment,” which are exactly the fields where most of the grass root innovations in experiential knowledge take place in Tibetan medicines. The creative innovations they develop may not be as effective as those used in high‐income settings, but often represent alternatives with excellent cost–benefit ratios adapted to their local contexts. National or subnational level of property right solutions needs to take this matter into account. In spite of the problems of ill use and low monitoring, India has offered the possibilities of inclusion of BtR innovations under the proprietary regimes within the confines of domestic market.

There are multiple rights that need to be balanced in Tibetan medicine, (a) the rights of traditional Tibetan physicians to practice and to access the medicinal plants; and (b) access to medicines by the public; (c) the rights for incremental innovations or new formulations developed from long term practice; (d) the protection from biopiracy threats from foreign patenting companies and importantly; and (e) the rights of the stakeholders when they share the tradition beyond geographical boundaries, etc. We have seen that the property approaches of China and India gives weight to selective stakeholders in different contexts. The Indian biomedical Pharmaceutical market have shown that national property regimes can boost the domestic industry and reach the global market (Kumar, 2002; Rasiah, 2002; Watal, 2000). The technological advance achieved by the Indian pharmaceutical industry owes much to the 1970 Patents Act^21^ and subsequent legalized reverse engineering of patented molecules. These reforms contributed many tangible benefits such as the creation of manufacturing capacities, lower medicine prices, and availability of medicines to the masses, hence more social benefits.

The national economies have their own mechanism of protection and a well‐structured institutional infrastructure to support Tibetan medicine as in China. Whether an economic or cultural property approach would benefit in this direction is not clear, but a sui‐generis mechanism should perform the dual responsibility of property right regime as a defensive mechanism against bio‐piracy threats and unfair exploitation and as a protective mechanism for grass root innovations and local resources in the context of increasing transnational patent applications and appropriations.

## ABOUT THE AUTHOR

Harilal Madhavan is Researcher at the Austrian Academy of Sciences’ Institute for Social Anthropology. He is an economist and intellectual property right expert in the ERC funded RATIMED (Re assembling Tibetan Medicine) Project based at the institute.

Harilal holds a PhD in economics from the Centre for Development Studies Thiruvananthapuram and JNU New Delhi, India (2012). His work specializes on the traditional pharmaceutical industry, intellectual property rights, natural resource economics, global public health, and the commercialization of traditional knowledge in South Asia. He has participated as a researcher in several national and international projects, including a large CNRS study based at EHESS (the subproject looked at the Intellectual property issues and Access and Benefit sharing mechanism in Traditional medicine in South India) in Paris and a Cluster of Excellence initiative at Heidelberg University.

## ENDNOTES

1 Both Tibetan medicine and *Sowa Rigpa* are used in different occasions of reference in the text. However, in transnational contexts, the term *Sowa Rigpa* is preferred as many Tibetans outside the Tibetan region do not regard themselves as Tibetan and prefer not to use the term *Tibetan medicine*.

2 A distinction can be made between the codified and non‐codified medical knowledge, in which the latter includes “folk,” “tribal,” or “indigenous” medicine, even though it shares blurred boundaries in geographical regions. The “folk” medicine is based on traditional beliefs; norms and practices based on centuries old experiences of trials and errors, successes and failures at the household level and the codified tradition consists of medical knowledge with sophisticated foundations expressed in thousands of manuscripts and canonical texts covering all branches of medicine like Chinese, Ayurveda, Siddha, Unani, and the Tibetan tradition.

3 Traditional Mongolian Medicine had developed by replacing many shamanistic practices with medical theories, techniques, and medication of Traditional Tibetan Medicine. From 16th century, for centuries, Tibetan Buddhism had influenced the daily life and the religious practice of the Mongolians as well as the medical education of the Mongolian physicians. Medical disputations and the exchange of ideas between Mongolian and Tibetan doctors stimulated the development of the Mongolian medicine.

4 SORIG herbal products are made by Men‐Tsee‐Khang based on the traditional Tibetan medical pharmacopoeia. These formulations are ancient, but sometime combinations are altered and do not contain any animal ingredient and are not tested on animals.

5 The excerpt is drawn with permission from the interview notes of Stephan Kloos with Men‐Tsee‐Khang officials during March April 2015.

6 The excerpt is from the interviews done by the author at Ladakh with Calum Blaikie during the month of June, 2015.

7 For detailed information, see the link: http://ec.europa.eu/health//sites/health/files/files/eudralex/vol-1/dir_2004_24/dir_2004_24_en.pdf

8 Many pharmaceutical giants like Pfizer, Novartis, Copaxone, Nexium, and Namenda—had a strong blow during 2012–15, due to the expiry of many pharmaceutical patents. In 2014, about $38.7 billion in pharma sales were likely be at risk due to the Loss of Exclusivity (LoE) due to off‐patents and by 2015, the figure was about $47.5 billion (nearly matching $54.7 billion losses during the 2012 patent cliff) (Gupta, 2015).

9 Regulations on Protection of Traditional Chinese Medicines—Promulgated by Decree No. 106 of the State Council of the People's Republic of China on October 14,1992 and came into effect from January 1,1993.

10 There are special precious formulas in Tibetan medicine namely *Rinchen Rilbu* or “precious pills.” These can take several months to gather the ingredients, detoxify them, and compound them into traditional supplements. Used as regenerative, preventive, and restorative to general health and serious illness alike, these medicines require proper guidance while taking these formulas.

11 For a detailed reading on this, please see Saxer (2013).

12 The information is from white paper (2008) protection and development of Tibetan culture 2008 (Information Office of the State Council of the People's Republic of China 25 September 2008, Beijing) link is: http://www.chinaun.org/eng/zt/xzwt/t521512.htm (accessed on 15, November 2015)

13 The department related parliamentary standing committee of Government of India on health and family welfare (2010) resulted in the *Forty sixth report on the Indian medicine central council (amendment) bill 2010*, Rajyasabha secretariat, New Delhi [online]. http://www.prsindia.org/uploads/media/Indian%20Medicine/SCR%20Indian%20Medicine%20CEntral%20Council%20Bill%202010.pdf [Accessed on January 19, 2016].

14 Government of India (various years) The Drugs and Cosmetic Act and Rules and later amendments, Ministry of health and family welfare, New Delhi.

15 Accessed from Press Information bureau, Ministry of Commerce and Industry, Government of India (http://pib.nic.in/newsite/PrintRelease.aspx?relid=98021 on March 20, 2016).

16 Hsiao (2007) mentioned that more than 70% of the doctors in Japan who were trained under the Western medical system prescribe *Kampo* extractions. Kampo is Japan's adaptation of TCM, which dates back to between the fifth and seventh centuries, owes its identity to the filtration or separation of underlying TCM theories.

17 See the details from the UNESCO portal, http://portal.unesco.org/en/ev.php-URL_ID=13039&URL_DO=DO_TOPIC&URL_SECTION=201.html

18 Convention for the Safeguarding of Intangible Cultural Heritage (CSICH), Paris 17 November 2003, (UNESCO Doc. MISC/2003/CLT/CH/ 14) entered into force in 2006. www.unesco.org/culture/ich/?pg=56 (retrieved on December 23, 2015).

19 The nine elements granted cultural heritage status include TCM approach to life and diseases, Chinese medicines diagnostic methods, techniques of preparing chinse medicinal material, traditional galenic preparation methods in Chinese medicine, acupuncture and moxibustion, medical culture of the Tongren tang Chinse pharmacy, medicinal culture of Huqingyu Tang Chinese pharmacy and Tibetan medicine.

20 China's response to the UNESCO questionnaire raising awareness on intangible cultural Heritage, available at www.unesco.org/culture/ich/doc/src/01179-EN.doc (accessed on January 20, 2016).

21 The Ayyangar Committee (1957–59) on Patents appointed by Government of India, studied the problems related to availability and affordability of modern medicines for public health requirements. In order to encourage local production of generic versions of new medicines and to make them available to the large number of poor people at a more affordable price, it recommended a non‐product patent regime in the field of pharmaceuticals. Consequently, India adopted a new Copyright Act in 1957, a new Trade and Merchandise Marks Act in 1958 and consequently a new Patents Act in 1970. (for details, available online, https://spicyip.com/wp-content/uploads/2013/10/ayyangar_committee_report.pdf) (accessed June, 22, 2017).

## Appendix 1 1

Details of Some important patents granted with reference to Tibetan medicine (google patents)

**Table:**

The patent holders	No of patents	Diseases/process	
Jigme Phuntsok	9	Diabetes, hair loss, kerato conjunctivitis, angina Pectoris, pharyngitis, thrombosis, peptic ulcers, acquired immunodeficiency syndrome, hepatitis B.	
SONG Yong heart	8	Hyperlipidemia, kerato conjunctivitis, influenza, cough, hypertension, chronic bronchitis, hemorrhoids, pharyngitis	
Lei Jufang	5	Acute and chronic sprain rheumatism, eye disease, hyperlipemia, women diseases.	
Zhangguo Xia,Li Jingtao, Chen Lijuan, Zhang Ying Shan, Wang Yanfeng	3	Bronchial asthma, fracture, hypertension	
Li Heng Fang, Chen Weiwu, Li Jingtao, Chen Lijuan	2	Diarrhea‐type irritability syndrome, fracture	
Song Qiang	1	Composition has anti‐inflammatory, analgesic, bactericidal, itching and other effects.	
Xuelian	1	Cervical spondylosis	
Li Xiaoqiang, Liu Yuan Wang	1	Acute and chronic sprain, rheumatism, rheumatoid aerosol	
Sang Jijia	1	Gout	
Yang Haixia, makin photo	1	Wound	
Jiangchuan Xiang, Wanqiang, bear Yan, Liu Min	1	Diabetes	
Dorje, Saisang Jie, Duojie La Dan, just let Nima	1	Formula and preparation process of Manuo‐series soup detoxification granule.	
Zhen Yuan, Chen Qin Li, R. Peng, W. Ping, D. Yubo	1	Quality inspection method of detoxification capsule formulation	
Ban Tengah measures	1	Heart cerebrovascular diseases and neurologic diseases,	
Fan autumn collar, Zhao Mingming	1	Method and compound and extraction eriophyton wallichii with antioxidant activity	
Wu Changchun	1	Facial mask	
Luoga	1	Diabetes	
Wang Jun	1	Bath lotion	
Measures Nigeria	1	Cough	
Sunfu	1	Haemorrhoid	
Ye Baolin, iron Shunliang	1	Cardiovascular disease	
